# Quantitative and qualitative characterization of Two PD-L1 clones: SP263 and E1L3N

**DOI:** 10.1186/s13000-016-0494-2

**Published:** 2016-05-18

**Authors:** Jacquelyn Smith, Mark D. Robida, Krista Acosta, Bharathi Vennapusa, Amita Mistry, Greg Martin, Alton Yates, H. James Hnatyszyn

**Affiliations:** Companion Diagnostics Pharma Services, Assay Development, Ventana Medical Systems Inc., Roche Tissue Diagnostics, 1910 E Innovation Park Drive, Tucson, AZ 85755 USA; Companion Diagnostics Pharma Services, Pathology Office, Ventana Medical Systems Inc., Roche Tissue Diagnostics, 1910 E Innovation Park Drive, Tucson, AZ 85755 USA; Companion Diagnostics, Project Management Office, Ventana Medical Systems Inc., Roche Tissue Diagnostics, 1910 E Innovation Park Drive, Tucson, AZ 85755 USA; Companion Diagnostics, Pharma Services, Clinical Services Management, Ventana Medical Systems Inc., Roche Tissue Diagnostics, 1910 E Innovation Park Drive, Tucson, AZ 85755 USA; Companion Diagnostics Pharma Services, CDx CAP/CLIA Lab Operations, Ventana Medical Systems Inc., Roche Tissue Diagnostics, 1910 E Innovation Park Drive, Tucson, AZ 85755 USA

**Keywords:** PD-L1, IHC, NSCLC, SP263, E1L3N

## Abstract

**Background:**

Programmed Death Ligand 1 (PD-L1) is an immune modulating protein expressed on the surface of various inflammatory cells, including T Cells, B Cells, dendritic cells, and macrophages. PD-L1 represents an important diagnostic target; expression of PD-L1 on the surface of tumor cells, or within tumor-associated immune cells, is an important predictor of likely response to targeted therapies. In this study, we describe the optimization of immunohistochemistry (IHC) assays using two PD-L1 antibodies (SP263 and E1L3N) and compare the performance of the optimized assays.

**Methods:**

Fully automated immunohistochemical assays were optimized for the VENTANA PD-L1 (SP263) Rabbit Monoclonal Antibody and the PD-L1 (E1L3N®) XP® Rabbit mAb using instruments and detection chemistries from Ventana Medical Systems, Inc. (“SP263 assay” and “E1L3N assay,” respectively). Tissue microarrays (TMAs) containing formalin fixed paraffin embedded (FFPE) non-small cell lung cancer (NSCLC) cases were used for the optimization and comparison staining. H scores were used for membrane scoring whereas percent positivity was used for tumor-associated immune cell scoring.

**Results:**

One-hundred NSCLC TMA case cores each stained with the SP263 and E1L3N assays were evaluated by two pathologists in a blinded study. Analysis of these specimens showed that the SP263 assay was more sensitive and had a wider dynamic range than the E1L3N assay. For sensitivity, many cases were found to be negative for membrane staining with the E1L3N assay, but had measurable staining with the SP263 assay. Dynamic range was demonstrated by the SP263 assay having well-distributed H scores while the E1L3N assay had a significantly higher proportion of cases clustered in the lowest H score bins. For tumor-associated immune cell staining, the two assays identified similar amounts of cells staining in each case, but the SP263 assay gave overall darker staining.

**Conclusion:**

Since PD-L1 status is important for targeted therapies, having a specific and accurate diagnostic test is crucial for identifying patients who could benefit from these treatments. Due to its staining intensity, scoring range, and pathologist preference, the SP263 IHC assay has been deemed superior to the E1L3N IHC assay. Future clinical utility remains to be determined.

**Electronic supplementary material:**

The online version of this article (doi:10.1186/s13000-016-0494-2) contains supplementary material, which is available to authorized users.

## Background

Tumor formation and persistence is a complex process involving a number of different cellular and subcellular aberrations that may or may not be mediated by abnormal cell signaling events. The tumor microenvironment plays a critical role not just in the formation of these malignancies, but also the maintenance, spread, and survival of the neoplasms. Components of the microenvironment that determine the stability of the tumor include the tumor cells themselves, the vasculature, and the tumor-associated immune cells. The tumor-associated immune cells play a critical, yet poorly defined, role in determining the survival or destruction of the tumor.

The role of the immune system in the formation, maintenance, or destruction of cellular malignancies is an emerging area of focus for cancer biologists and clinicians [1–5]. Although the concept itself is not new, the mechanisms mediating these processes have yet to be elucidated. Some components of the innate immune system, including natural killer cells, have been implicated in angiogenic events supporting tumor formation and growth [6]. However, other components of the immune system, including macrophages and lymphocytes, have been associated with anti-tumorigenic activities targeting these neoplasms [7, 8]. Furthermore, the type and extent of the tumor-associated immune cells has been associated with variable clinical outcomes, and therefore certain immune-specific antigens can represent attractive clinical and diagnostic targets [9].

Most therapies targeting immune modulating antigens either promote an immune response, or inactivate immune-inhibitory mechanisms. Proteins within a certain T Cell associated pathway, the PD-L1/PD-1 pathway, tend to be commonly targeted. Programmed Death Ligand 1 (PD-L1) is an immune modulating protein expressed on the surface of various inflammatory cells, including T Cells, B Cells, dendritic cells, and macrophages [10]. Overexpression of PD-L1 within normal inflammatory cells, as well as ectopic expression on the surface of tumor cells, has been associated with tumor persistence as a result of an ablated immune response [11, 12]. Expression of PD-L1 on the surface of tumor cells inactivates primed CD8+ T Cells by binding to its high affinity receptor, PD-1. This immune inhibiting effect shuts down the machinery programmed to destroy the tumor cells.

Several directed therapies targeting either the receptor (PD-1) or its ligand (PD-L1) are in various phases of clinical trials [13]. By binding to either PD-1 or PD-L1, these monoclonal antibodies effectively prevent PD-L1-induced activation of the PD-1 pathway, and shut down the immune-inhibiting function of PD-L1. As a target of therapy, PD-L1 represents an important diagnostic target; expression of PD-L1 within the tumor-associated immune cells or on the surface of tumor cells is an important predictor of likely response to these targeted therapies. Therefore, a diagnostic test that specifically and accurately detects PD-L1, with sufficient analytic sensitivity, is critically important in order to identify patients likely to benefit from these treatments.

Immunohistochemical tests are relatively inexpensive, quickly performed, and widely accessible in most clinical markets. Additionally, assaying for the protein itself provides direct diagnostic data that is more likely to correspond to a clinical benefit than detecting non-therapeutic targets, such as DNA or RNA. With a multitude of commercially available antibodies and staining platforms available, identifying a single clone and associated assay is important in order to standardize the diagnostic modality used. This report describes the optimization and comparison of two PD-L1 clones with respect to their analytical performance and clinical applications.

## Methods

### Specimens

TMAs containing formalin fixed paraffin embedded (FFPE) non-small cell lung cancer (NSCLC) cases (LC1923) were procured from US Biomax, Inc. (Rockville, MD, USA) and were used for optimizing the PD-L1 immunohistochemistry (IHC) assays and performing the assay comparison staining. FFPE sections (4 μm) were provided by the vendor on positively charged slides. A board certified pathologist reviewed H&E stains of the specimens and all were deemed acceptable for use.

### PD-L1 antibody clones

Two anti-PD-L1 antibodies were examined, clones SP263 (Ventana Medical Systems, Tucson, AZ) and E1L3N (Cell Signaling Technologies, Danvers, MA). The SP263 rabbit monoclonal was generated with a synthetic peptide derived from the C-terminus of human PD-L1 protein. The E1L3N rabbit monoclonal was produced by immunizing with a synthetic peptide corresponding to residues near the C-terminus.

### Automated PD-L1 immunohistochemistry staining procedure optimization

Immunohistochemical staining was carried out on the VENTANA BenchMark ULTRA automated staining platform using the OptiView detection kit (VMSI, Catalog No. 760–700). The manufacturer recommend dilution of the E1L3N clone for a manual overnight staining protocol produced suboptimal staining quality on the automated staining platform. Therefore, the E1L3N clone was re-optimized for direct comparison to the SP263 clone. During development and optimization of the SP263 and E1L3N assays, antigen retrieval, primary antibody concentration, and primary antibody diluent were examined.

Antigen retrieval was optimized by evaluating a variety of cell conditioning buffers and incubation times. Absence of cell conditioning was investigated and determined to yield unacceptable staining. Cell conditioning 1 (CC1) buffer (VMSI, Catalog No. 950–224) is a TRIS based buffer of alkaline pH. This was tested using a 32 min incubation both with and without Protease 3 (VMSI, Catalog No. 760–2020) for 4 min, and a 64 min incubation of only CC1. Cell conditioning 2 (CC2) buffer (VMSI, Catalog No. 950–223) is a citrate based buffer with an acidic pH. This buffer was evaluated using a 32 min incubation time. Finally, a more potent enzymatic antigen retrieval reagent, Protease 1 (VMSI, Catalog No. 760–2018), was evaluated for 4 min of incubation time. Of these conditions, 64 min of CC1 at standard temperature was superior to the other conditions tested and was selected as the antigen retrieval parameter for the final assays.

The intensity of staining at different concentrations of the anti-PD-L1 antibodies was evaluated by the pathologist to select the ideal titer. An automatically dispensed concentration of each clone was selected based on the pathologist’s interpretation of relevant positive staining and non-specific background reactivity.

Finally, five diluents (VMSI proprietary) were screened and evaluated to optimize staining quality. The choice of diluent potentially impacts not only positive staining intensity, but also background staining and antibody stability. The diluent that resulted in the most ideal signal to noise ratio was selected for the final assay.

Both clones performed best with the same assay conditions, although the optimal antibody concentrations differed (Table 1).

**Table 1:** Description of Anti-PD-L1 Antibodies and Assay Conditions Examined

Clone	Vendor	Species	Working Concentration	Instrument	Detection	Cell Conditioning	Diluent	Primary Antibody Time/Temp	Hematoxylin/Bluing
SP263	Spring	Rabbit	1.25 μg/mL	Benchmark Ultra	OptiView	64' CC1	0.05 M Tris-HCI with 1 % carrier protein, and 0.10 % ProClin 300, a preservative	16'/36 °C	4'/4'
E1L3N	CST	Rabbit	17.50 μg/mL	Benchmark Ultra	OptiView	64' CC1	0.05 M Tris buffered saline, 0.01 M EDTA, 0.05 % Brij-35 with 0.3 % carrier protein and 0.05 % sodium azide, a preservative	16'/36 °C	4'/4'

### Final automated PD-L1 immunohistochemistry staining procedures

Antigen recovery was conducted for 64 min with CC1 buffer. Slides were incubated with a dilution of the stock primary antibodies in a dilution buffer for 16 min at 36 °C. Stock antibody refers to the concentration at which the antibody was provided to Ventana by the manufacturer. The SP263 assay was used at 1.25 μg/mL and the E1L3N assay was used at 17.50 μg/mL in the final optimized assays. As a negative reagent control, specimens were incubated with a rabbit monoclonal negative control antibody under the same conditions.

The primary antibodies were detected using the OptiView detection kit. Enzymatic detection of anti-PD-L1 antibodies was accomplished with a secondary goat anti-mouse and anti-rabbit IgG conjugated to HQ, followed by an anti-HQ conjugated to HRP. Chromogen was deposited by a reaction with hydrogen peroxide in the presence of diaminobenzidine (DAB) and copper sulfate. The secondary antibody, HRP multimer, and all chromogen reagents were applied at the instrument’s default times.

### Slide evaluation

Serial sections of a commercial TMA (LC1923) containing one hundred NSCLC cases were stained with the SP263 and E1L3N assays. Two board certified pathologists evaluated one hundred NSCLC cases. Interpretation of stains included assessment of tumor cell staining for membrane localization as well as percent positivity of the tumor-associated immune cells. Stained tissues were scored on a four point scale (0, 1, 2, and 3) for membrane tumor staining. Dark brown membrane staining was scored a 3, moderate brown membrane staining a 2, and light tan to brown membrane staining intensity a 1 (no membrane staining was scored a 0). The percentage of cells staining positively at each intensity level was recorded. In tumor samples, only viable malignant cells were scored.

H scores, which combine components of staining intensity with the percentage of positive cells, were calculated. H scores have a value ranging from 0 to 300 and are defined as:

- 1 * (percentage of cells staining at 1+ intensity)
- +2 * (percentage of cells staining at 2+ intensity)
- +3 * (percentage of cells staining at 3+ intensity)
- = H score.

Tumor-associated immune cell staining was measured as percent positive cells infiltrating the tumor at any intensity of stain.

During evaluation, the pathologists were blinded to the assays performed on each array.

The two pathologists independently participated in a survey to assess the qualitative performance of each assay. Questions were answered on a scale of 1 to 10, with 1 being very much disliked and 10 being very much liked. Areas assessed included ease of interpreting membrane staining in the tumor and in the tumor-associated immune cells, the ability to distinguish tumor staining from tumor-associated immune cell staining, and signal to noise ratio. The survey also focused on overall assay aesthetic preference and the ability of the assay to serve as a companion diagnostic in a clinical setting. The results from the survey are summarized in Table 2.

**Table 2:**
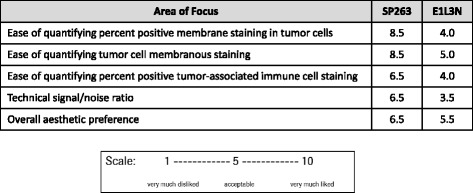
PD-L1 Pathology Evaluation Survey and Results

### Slide imaging

Images of stained slides were captured on the Aperio ScanScope® XT system. Slides were scanned at 20X and the resulting images were used for photomicrographs.

### Data analysis

Data analyses were performed using standard available functions in Excel. The scientists performing the data analyses were blinded to the identity of the assays until all analysis was completed.

Assay specificity for the SP263 and E1L3N assays was determined by noting cell types stained and the subcellular localization of the stain. Assay sensitivity and range were examined by comparing calculated H scores. Concordance between assays (inter-assay) and concordance between pathologists (inter-pathology) were evaluated. Inter-assay correlation was gauged by comparing a single pathologist’s H scores for membrane staining in the tumor along with the percent positivity of the tumor-associated immune cells for the SP263 and E1L3N PD-L1 assays. Inter-pathologist correlation was determined by comparing each pathologist’s H scores for membrane staining in the tumor along with the percent positivity of the tumor-associated immune cells for the same PD-L1 assay. Tumor-associated immune cell staining was analyzed by comparing the percentages of cells that stained for each assay.

The pathologists’ qualitative assay survey results were quantified using the numerical answers given to each question and resulted in an overall assay preference score.

## Results

### Assay optimization

The Spring Bioscience anti-PD-L1 antibody clone SP263 and the CST anti-PD-L1 antibody clone E1L3N were optimized for use in a fully automated immunohistochemical assay on the VENTANA BenchMark ULTRA staining platform with the OptiView DAB IHC Detection Kit. The assay was optimized for detection of PD-L1 expression in non-small cell lung cancer (NSCLC). Parameters evaluated included antibody concentration, antibody diluent, cell conditioning time and buffer, and antibody incubation time and temperature. The optimized assay conditions for each antibody were identical, with the exception of the optimal antibody concentration (Table 1).

### Qualitative assay evaluation

NSCLC specimens stained with the SP263 and E1L3N assays were evaluated by two pathologists in a blinded study. Staining was evaluated on a 1–10 scale for quality of PD-L1 membrane staining in the tumor, as well as for PD-L1 staining in the tumor-associated immune cells. The ease of quantifying both the staining itself and the percentage of positive cells were evaluated, as was the signal to noise ratio, and overall assay aesthetics. The results are summarized in Table 2.

**Fig. 1:**
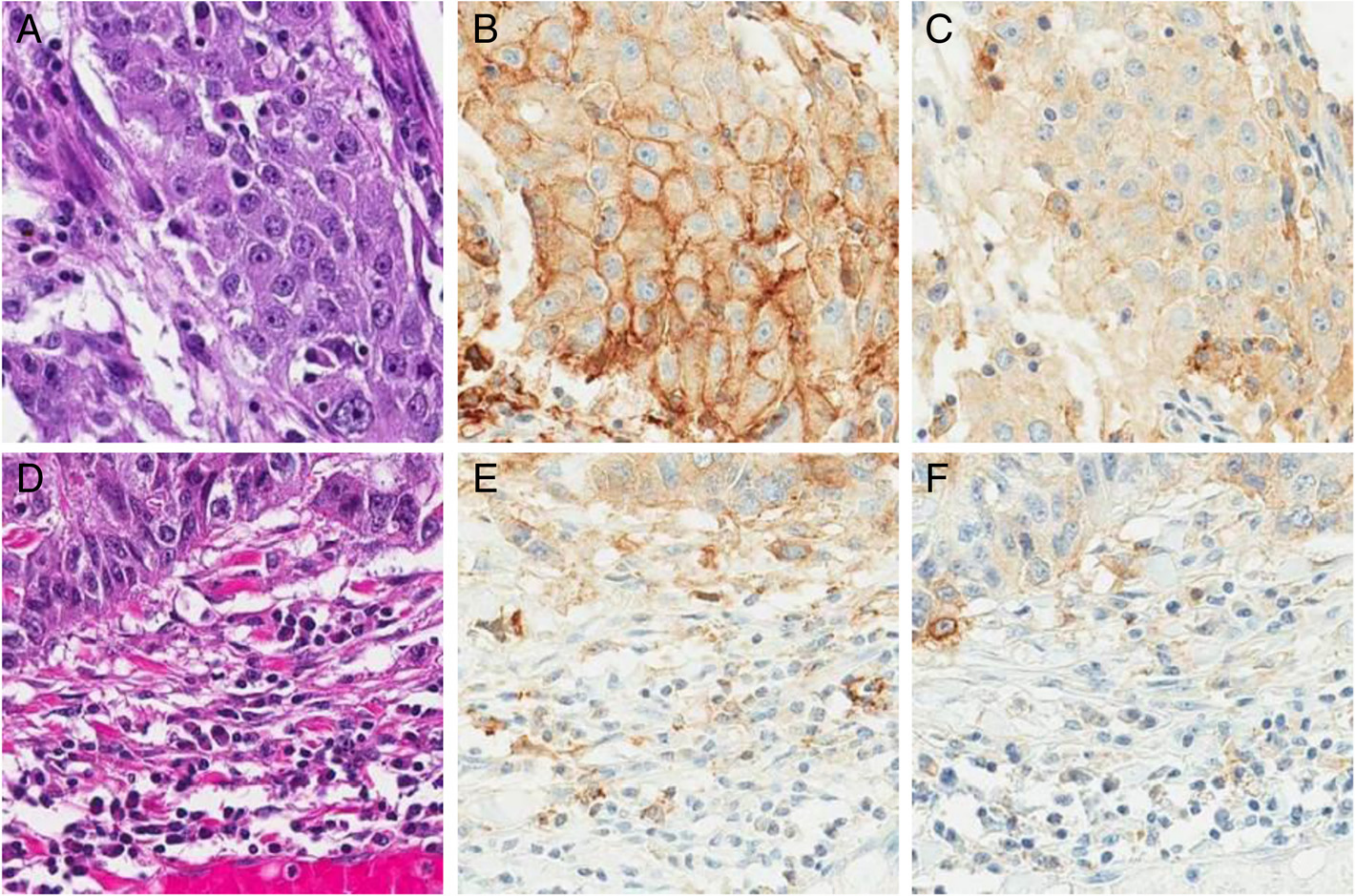
Immunohistochemical Staining of PD-L1 on NSCLC **a** H&E of NSCLC case D4 (20X magnification). **b** SP263 Assay demonstrating membrane and cytoplasm staining of tumor cells (20X magnification). **c** E1L3N Assay demonstrating membrane and cytoplasm staining of tumor cells (20X magnification). **d** H&E of NSCLC case A15 (20X magnification). **e** SP263 Assay demonstrating staining of tumor-associated immune cells (20X magnification). **f** E1L3N Assay demonstrating staining of tumor-associated immune cells (20X magnification)

The SP263 assay received significantly higher scores from the pathologists for identification of membrane staining, as well as for ease of quantifying the percentage of positive tumor cells with membrane staining and the percentage of positive tumor-associated immune cells. The E1L3N assay was deemed unacceptable for ease of quantifying the percent of positive staining in tumor cell membranes and in the tumor-associated immune cells. Although both assays stained similar numbers of inflammatory cells, the darker staining of the SP263 assay allowed for easier quantification (Fig. 1). The SP263 assay received a significantly higher score for signal to noise ratio than the E1L3N assay, indicating that it was cleaner, with specific staining more easily differentiated from any non-specific background staining (Table 2). Both assays received similar scores for overall aesthetic preference, with the SP263 assay slightly preferred.

### Assay specificity

The specificity of each assay was assessed by observing the types of cells staining in the NSCLC cases and by observing the subcellular localization of the staining and comparing with previously published reports. Both assays stained the cytoplasm and membrane of tumor cells (Fig. 1b, c) as well as cells in the tumor-associated immune cells (Fig. 1e, f), consistent with previous reports [14, 15]. As expected, non-neoplastic cells were negative for PD-L1 staining with both assays, yielding good signal to noise ratios.

### Assay sensitivity

Analysis of the one hundred NSCLC cores found that the SP263 assay was more sensitive than the E1L3N assay. Twenty-nine cases found to be negative for membrane staining by the E1L3N assay were found to have measurable anti-PD-L1 staining with the SP263 assay (Additional file 1: Table S1). In all twenty-four cases positive by both assays, the SP263 assay yielded higher H scores for membrane anti-PD-L1 staining.

**Fig. 2:**
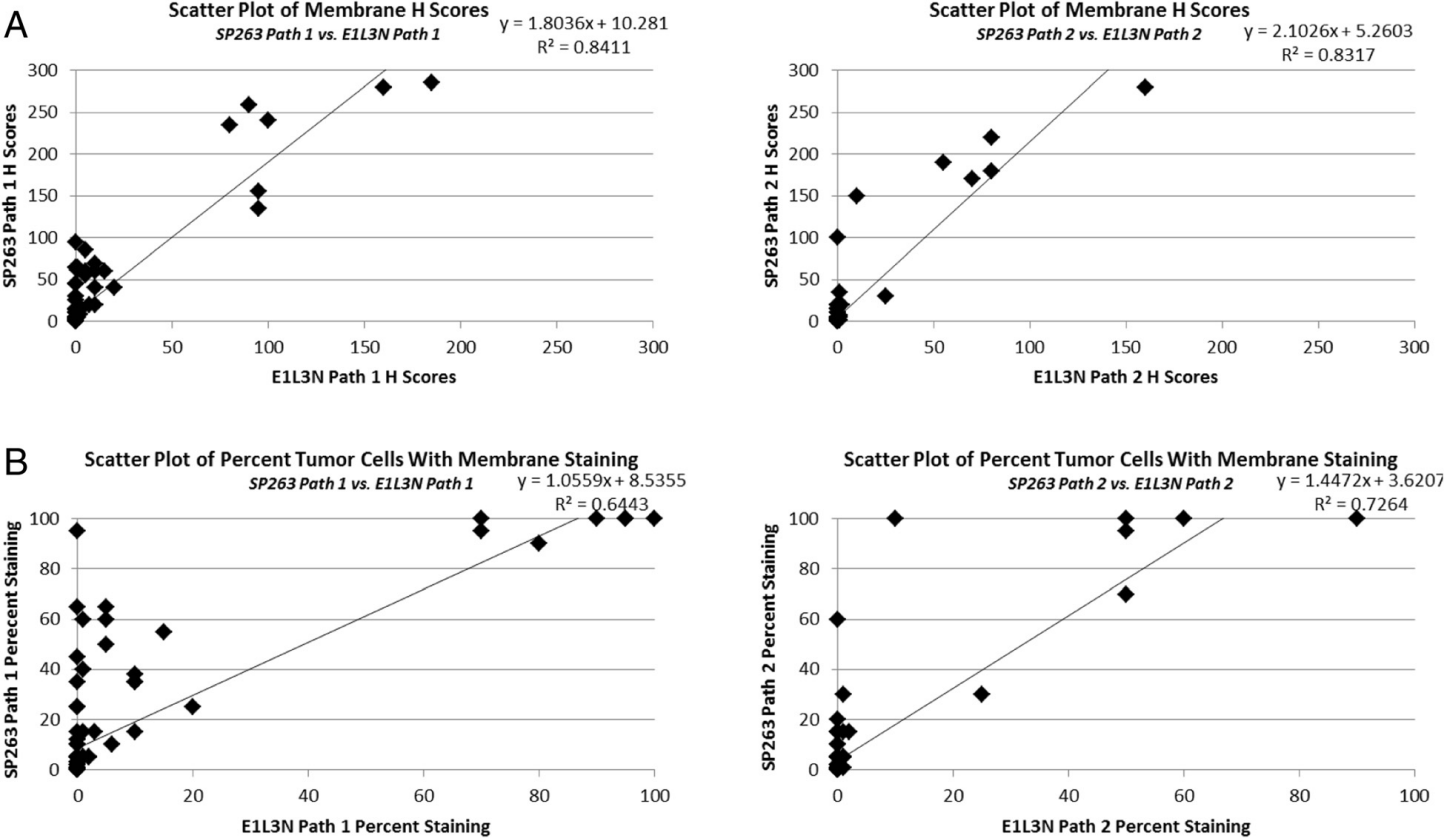
Analysis of PD-L1 Assay Sensitivity **a** Scatter plots comparing membrane H scores for each assay from pathologist 1 and 2. **b** Scatter plots of the percent of tumor cells with membrane staining for each assay from pathologist 1 and 2

When the membrane H scores for each case are plotted against each other on a scatter plot the slope of the best fit line is greater than 1 (Fig. 2a), indicating a higher sensitivity for the SP263 assay. This trend was seen by both evaluating pathologists. Scatter plots of the percent of tumor cells with membrane staining identified by each assay indicate that the SP263 assay identifies more positive cells than the E1L3N assay, particularly at the lower intensity levels (Fig. 2b). Since H scores are a product of both the percentage of cells staining and the intensity at which the cells stain, and the SP263 assay identifies more cells staining at greater intensity, the H scores tend to be higher for the SP263 assay than the E1L3N assay on the same cases. This can be seen by examination photomicrographs of cases stained with each assay (Fig. 1b, c).

### Assay range

**Fig. 3:**
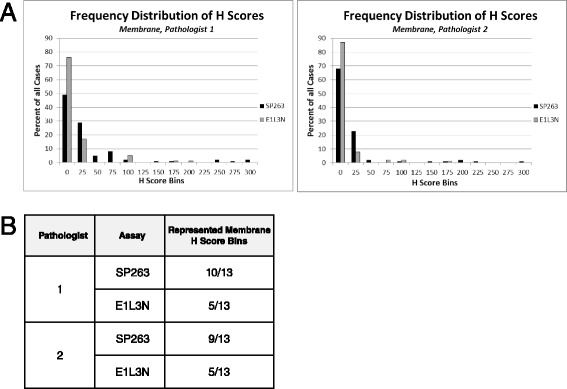
Analysis of PD-L1 Assay Range of Tumor Area **a** Histograms of tumor membrane H scores, binned from 0 to 300 in increments of 25, and the percentage of cases in each of the bins from pathologist 1 and 2. **b** A table showing how each assay was represented across membrane H score bins from pathologist 1 and 2

Analysis of the one hundred NSCLC cores also found that the SP263 assay had a wider dynamic range than the E1L3N assay. Tumor membrane H scores were binned using increments of 25, and the percentage of cases in each bin was used to generate histograms (Fig. 3a). Membrane H score distribution for the SP263 assay ranged from the 0 bin to the 300 bin, whereas the membrane H scores for the same samples with the E1L3N assay ranged from the 0 bin to the 200 bin. While the SP263 assay had well-distributed scores, the E1L3N assay had a significantly higher proportion of cases clustered in the lowest scoring bins. In addition, the SP263 assay had cases fall into a larger number of bins than the E1L3N assay (Fig. 3b). These observations were consistent between the two pathologists.

**Fig. 4:**
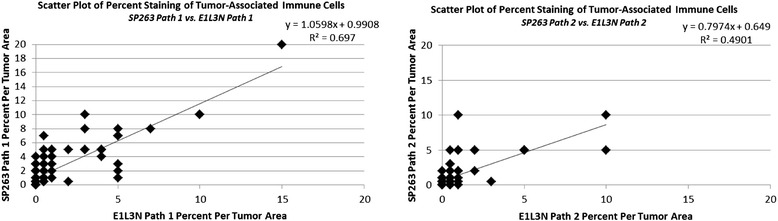
Analysis of PD-L1 Assay Range of Tumor-Associated Immune Cells Scatter plots comparing percent staining of tumor-associated immune cells for each assay from pathologist 1 and 2

For tumor-associated immune cell staining, the SP263 and E1L3N assays had similar ranges (Fig. 4, Additional file 2: Table S2). Both assays typically had staining in 0–10 % of the immune infiltrate, with only a single case for each assay staining a higher percentage. Although the two assays identified similar amounts of cells staining in each case, the SP263 assay gave an overall darker staining (Fig. 1e, f). This allowed for easier quantification of staining in the tumor-associated immune cells using the SP263 assay, and contributed to the pathologists’ preference for this assay.

### Inter-pathologist correlation

**Fig. 5:**
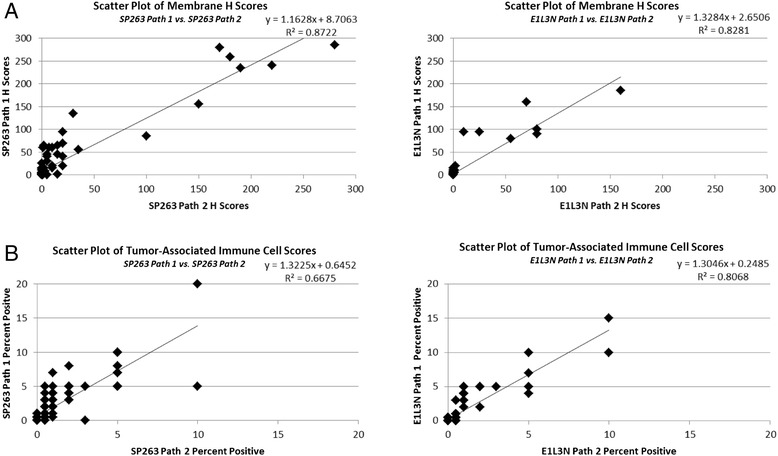
Inter-pathologist Correlation of PD-L1 Assays (**a**) Scatter plots comparing membrane H scores for each assay from pathologist 1 and 2. **b** Scatter plots comparing percent positivity in tumor-associated immune cells for each assay from pathologist 1 and 2

To determine the level of inter-pathologist correlation when scoring each assay, the individual pathologists’ H scores for membrane staining in the tumor and percent positive cells in the tumor-associated immune cells were compared. Both the SP263 assay (R^2^ > 0.87) and the E1L3N assay (R^2^ > 0.82) had high inter-pathologist correlation for membrane tumor staining scores (Fig. 5a). The range of scores for the E1L3N assay is significantly compressed relative to the SP263 assay. These results indicate that the tumor cell staining generated by both assays can be scored reproducibly by different pathologists, with the SP263 assay producing the more concordant results. Both assays had lower inter-pathologist correlation (SP263 R^2^ > 0.66, E1L3N R^2^ > 0.80) for percent of positively staining cells in the immune infiltrate (Fig. 5b). The E1L3N assay had stronger inter-pathologist correlation, but had almost twice as many cases with no staining as compared to the SP263 assay (Additional file 2: Table S2).

## Discussion

Recent studies have linked PD-L1 expression in tumors with response to immunotherapies targeting PD-L1 or its receptor PD-1 [15–18]. These findings highlight the necessity of a specific, robust, fully-automated PD-L1 IHC assay to be used in patient selection for clinical trials and to identify additional tumor types that may benefit from targeted PD-L1/PD-1 immunotherapy.

Fully automated immunohistochemical assays were optimized for the VENTANA PD-L1 (SP263) Rabbit Monoclonal Antibody and the PD-L1 (E1L3N®) XP® Rabbit mAb using instruments and detection chemistries from Ventana Medical Systems, Inc. (“SP263 assay” and “E1L3N assay,” respectively). Both assays stained PD-L1 in the tumor membrane and cytoplasm, and also in the tumor-associated immune cells, consistent with previous results with other PD-L1 clones [14, 15]. The tumor areas stained and subcellular localization pattern within tumor cells was consistent across both assays. Both assays demonstrated high signal to noise ratio, and staining was not observed in normal lung tissue or in cancer-adjacent stromal regions. Taken together these data suggest that both the SP263 and E1L3N assays are highly specific for PD-L1. Both assays also generated scores in the tumor cell membrane that were highly reproducible from pathologist to pathologist, a characteristic required for future use as a diagnostic assay. Pathologist to pathologist correlation for percent positive tumor-associated immune cells was lower for both assays, indicating that consistent scoring of the percent positivity in the tumor-associated immune cells is more difficult than H scoring of tumor cells. Low percent positivity means that small changes in estimated percent staining can have a large effect on the correlation of the data.

Although both assays stained with a similar pattern and stained similar percentages of tumor cells and tumor-associated immune cells, the SP263 assay was highly preferred by both pathologists when blinded to the identity of each assay in the qualitative survey. The SP263 assay yielded significantly darker staining in the tumor cells and tumor-associated immune cells compared to the E1L3N assay. The difference in staining intensity allowed for easier quantification of the staining in the tumor membrane and for easier quantification of percentage of cells staining in the tumor-associated immune cells for the SP263 assay. The average H score for the membrane tumor cell staining was higher for the SP263 assay in all cases that were positive by both assays, and the SP263 assay generated a much larger range of scores than the E1L3N assay, which was clustered towards the low end of the scoring range. In addition, PD-L1 staining was detected by the SP263 in many NSCLC cases that were negative by the E1L3N assay, suggesting that the SP263 assay has higher sensitivity. The overall weaker staining using the E1L3N assay likely contributes to a higher likelihood of false negatives, especially in cases where only focal staining is present.

Definitions of PD-L1 positivity vary from study to study and from cancer type to cancer type, and include different thresholds of expression in either tumor cells and/or the tumor-associated immune cells [15–17, 19, 20]. The ideal IHC assay for detection of PD-L1 expression should allow for a wide range of scores in both tumor cells and tumor-associated immune cells; using future clinical outcome data the threshold of PD-L1 clinical positivity could be further refined. Based on its staining characteristics, the SP263 assay is superior for detecting PD-L1 expression in both tumor cells and tumor-associated immune cells, and is promising for use as both a diagnostic tool and a means of patient stratification for immunotherapy. The clinical utility of this assay needs to be verified in clinical studies.

## Conclusions

In conclusion, the SP263 assay is superior for detecting expression of PD-L1 in tumor cells and/or tumor-associated immune cells, when compared to the E1L3N assay. The SP263 assay not only demonstrated darker staining intensity in tumor cells and tumor-associated immune cells, but also generated a greater scoring range which suggests higher sensitivity. The blinded board-certified pathologists who scored the assay expressed, in their qualitative survey, a preference for the SP263 assay. Since PD-L1 status is important for targeted therapies, having a specific and accurate diagnostic test is crucial for identifying patients who could benefit from these treatments. Although SP263 is an analytically superior assay to the others tested, evaluating its predictive claim to any specific therapy can only be accomplished during the course of a controlled clinical trial.

## Additional files

Additional file 1: Average Membrane H Scores for 100 NSCLC Cases Stained with the SP263 Assay and the E1L3N Assay. (PPTX 86 kb) Additional file 2: Average Percent Positive Tumor-Associated Immune Cells for 100 NSCLC Cases Stained with the SP263 Assay and the E1L3N Assay. (PPTX 97 kb)

## Abbreviations

CC1: cell conditioning 1 buffer
CC2: cell conditioning 2 buffer
DAB: diaminobenzidine
DNA: deoxyribonucleic acid
FFPE: formalin fixed paraffin embedded
H&E: hematoxylin and eosin stain
HQ: hydroquinone
HRP: horseradish peroxidase
IHC: immunohistochemistry
NSCLC: non-small cell lung cancer
PD-1: programmed cell death-1
PD-L1: programmed death-ligand 1
RNA: ribonucleic acid
