# Supplementary figures and images for: Quantitative and qualitative characterization of Two PD-L1 clones: SP263 and E1L3N
Source: Diagn Pathol. 2016 May 18;11:44. doi: 10.1186/s13000-016-0494-2 (PMC4870735; doi:10.1186/s13000-016-0494-2)

## Slide 1
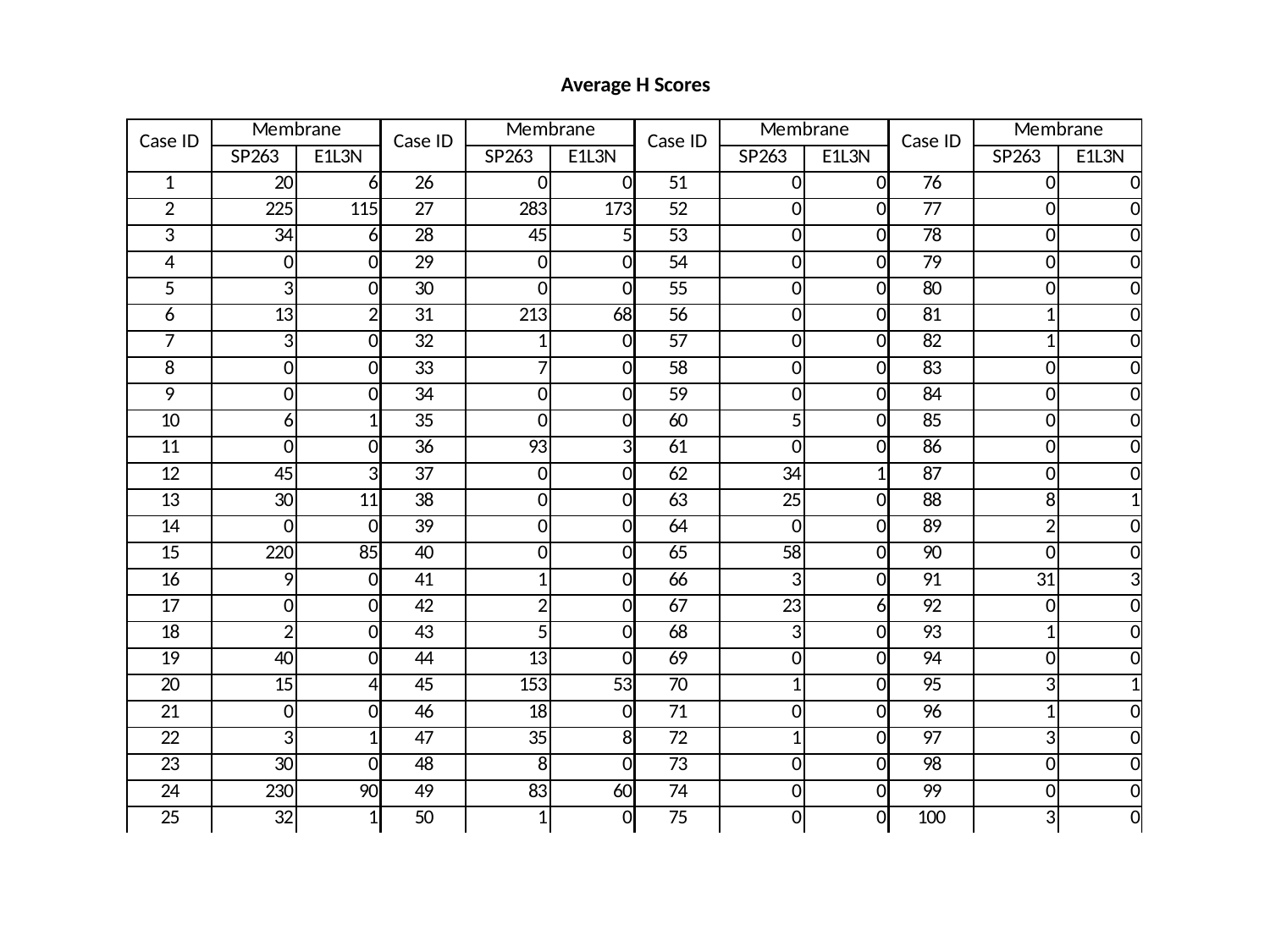

Average H Scores

Supplement: Additional file 1: — Average Membrane H Scores for 100 NSCLC Cases Stained with the SP263 Assay and the E1L3N Assay. (PPTX 86 kb) [file 13000_2016_494_MOESM1_ESM.pptx]

## Slide 1
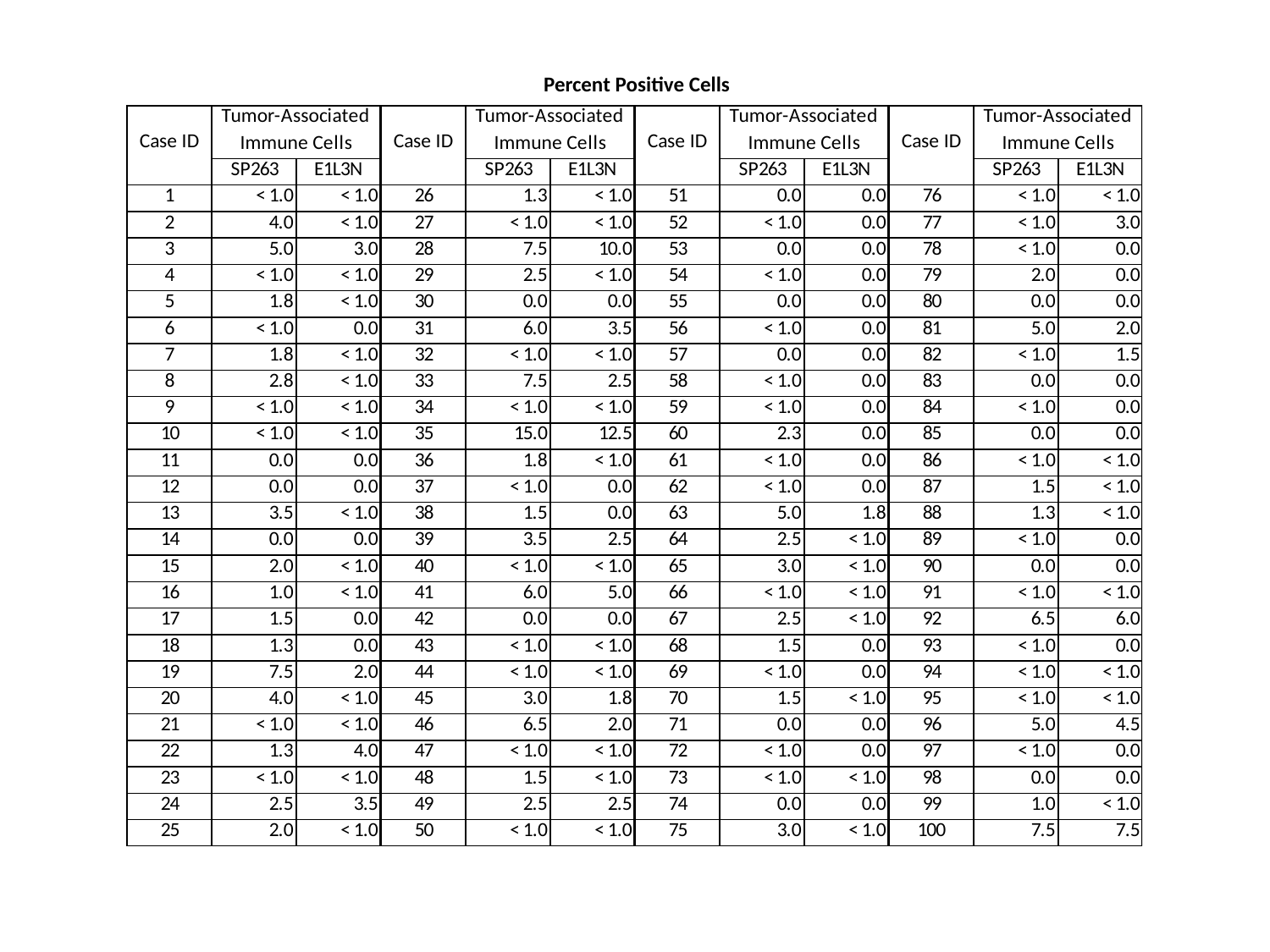

Percent Positive Cells

Supplement: Additional file 2: — Average Percent Positive Tumor-Associated Immune Cells for 100 NSCLC Cases Stained with the SP263 Assay and the E1L3N Assay. (PPTX 97 kb) [file 13000_2016_494_MOESM2_ESM.pptx]
